# Migraine and Its Treatment from the Medicinal Chemistry Perspective

**DOI:** 10.1021/acsptsci.3c00370

**Published:** 2024-03-16

**Authors:** Ezgi Pehlivanlar, Simone Carradori, Rahime Simsek

**Affiliations:** † Department of Pharmaceutical Chemistry, Faculty of Pharmacy, Hacettepe University, 06100 Ankara, Turkey; ‡ Department of Pharmacy, University “G. d’Annunzio” of Chieti-Pescara, 66100 Chieti, Italy

**Keywords:** Migraine, pathophysiology, acute/chronic treatment, triptans, ditans, CGRP receptor antagonists

## Abstract

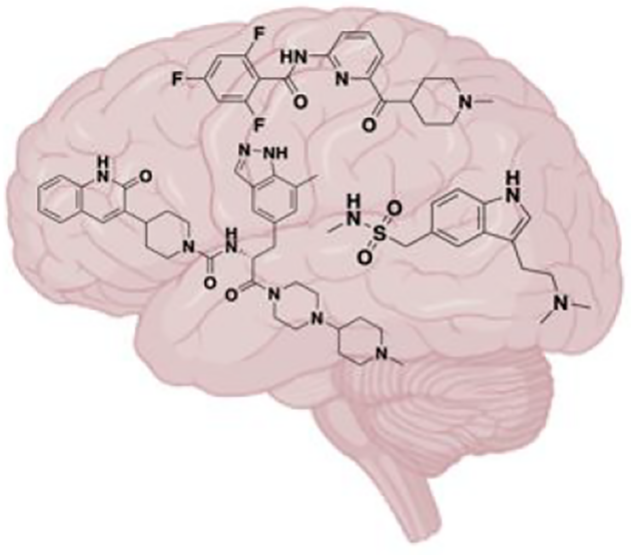

Migraine is a disease of neurovascular origin that affects the quality of life of more than one billion people and ranks sixth among the most common diseases in the world. Migraine is characterized by a moderate or severe recurrent and throbbing headache, accompanied by nausea, vomiting, and photo-phonophobia. It usually starts in adolescence and is twice as common in women as in men. It is classified as with or without aura and has chronic or acute treatment types according to the frequency of occurrence. In acute treatment, analgesics that relieve pain in the fastest way are preferred, while there are different options in chronic treatment. While non-specific methods were used in the treatment of migraine until the 1950s, triptans, ditans, and CGRP-receptor-dependent therapies (monoclonal antibodies and gepants) started to be used in the clinic more recently. In this Review, we focus on the synthesis, side effects, and pharmacological and pharmacokinetic properties of FDA-approved drugs used in acute and preventive-specific treatment of migraine.

Migraine is a common disease affecting people of all ages, characterized by symptoms such as nausea, vomiting, and photo-phonophobia accompanying moderate to severe recurrent headache attacks of neurovascular origin.^1^ It ranks sixth among the most common diseases.^2^ The quality of life of more than 1 billion people in the world decreases due to migraine.^3^ According to data from the World Health Organization, migraine usually begins in adolescence and mostly affects individuals between the ages of 35 and 45. It is seen twice as often in women as in men due to hormonal changes.^4^ The period of migraine can vary between 4 and 72 h. Although various mechanisms have been suggested for the pathophysiology of migraine, there is no definitive treatment method because the exact cause is not known. Therefore, one of the subjects of drug research and development studies is migraine.^5^ Because of the symptoms that occur during a migraine attack, this disease significantly reduces the patient’s quality of life. Even if only one of these symptoms occurs, the need to access available medications during a migraine attack increases. Suicidal ideation may occur in patients with migraine attacks due to the intense pain they feel.^6^

Migraine is characterized by severe headache and may occur in the cortical, subcortical, and brainstem regions. Headache may cause autonomic, cognitive, and affective symptoms over time.^7^ Factors such as light, noise, and smell trigger migraine attacks in many migraine patients. Lack of sleep or too much sleep, skipping meals, the stress of daily life, intense exercise, and hormonal changes are other factors that trigger migraine attacks because they cause changes in the normal activity of the hypothalamus^8^ (Figure 1). Additionally, one-third of patients with migraine experience an abnormal sensitivity to light (photophobia) before the onset of migraine headache, and this sensitivity is exacerbated by light.^9^ In noisy environments, the inability to distinguish and understand sounds and hypersensitivity to auditory stimuli cause discomfort in the patient and a sense of avoidance from these environments. This behavior is also called phonophobia.^10^ Odor aversion, referred to as osmophobia, has been suggested to trigger tension-type headache in studies conducted in patients with migraine, and it is considered as one of the differential diagnoses of migraine.^11^ In addition to medical treatment, eliminating these unfavorable conditions, either from the patient themselves or from the environment, can also contribute to the reduction or alleviation of migraine attacks. Migraine progresses in four phases: premonitor, aura, headache, and postdrome.^12^

**Figure 1:**
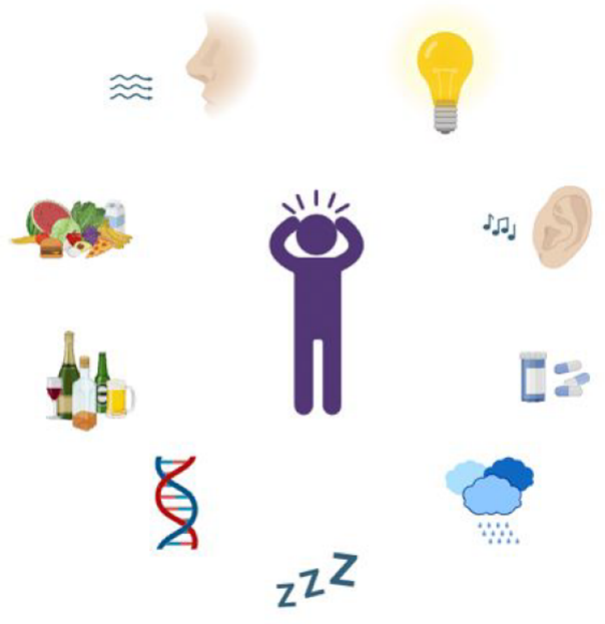
Migraine triggers.

## 1.1 Premonitor Phase

The premonitor phase begins 72 h before the headache phase.^13^ In the premonitory phase, the most commonly reported symptoms are fatigue, photophobia, and phonophobia, but cognitive changes, yawning, abnormal hunger, thirst, irritability, and difficulty concentrating are also seen.^14^ The hypothalamus, which is responsible for the body’s homeostasis and sleep cycle, endocrine, and autonomic regulation, is connected to the cortical and subcortical regions of the brain. Hypothalamic hyperactivation occurs during the premonitor phase.^15^ Neurotransmitters such as dopamine, orexin, vasopressin, and somastatin are released.^16^ In 1899, Gowers described the premonitor phase as drowsiness. In 1980, it was joined by Blau as “complete migraine”. Initially, considering the role of the hypothalamus in the premonitory phase, it was contradictory to the trigeminovascular system mechanism of migraine.^17^

## 1.2 Aura Phase

The aura phase is a phase in which waves containing visual, auditory, speech, and/or motor symptoms precede the headache.^18^ The most common symptom is visual aura. It is seen in one-third of patients with migraine. The pathophysiology of the aura is related to the depolarization of the cortex and the creation of a temporary wave as a result of neurological symptoms.^19^ Aura phase lasting more than 1 h or dramatic increases in aura episodes should be investigated, and precautions should be taken against the risk of ischemic stroke.^20^ Women are at increased risk of stroke due to the use of oral contraception. In the aura phase, non-steriodal anti-inflammatory drugs (NSAIDs) are recommended to prevent the headache phase, not to treat the aura. In case of failure, triptans are recommended.^12,21^

## 1.3 Headache Phase

In the headache phase, moderate or severe headache, usually occurring on one side of the head, may also be accompanied by pain in the face and neck,^22^ and vomiting may accompany the headache in this phase.^23^ This attack phase of migraine may last for hours. Starting from the supraorbital region, the pain intensifies toward the temples and eyebrows. Pain is triggered by intense odors, bright light, noise, and stress. It may cause gastrointestinal side effects such as constipation.^19^ In the headache phase, activation of the trigeminovascular pathway occurs. The trigeminovascular nerves are innervated and sensory information is processed to higher cortical areas before being transmitted to the contralateral thalamus.^24^

## 1.4 Postdrome Phase

The postdrome phase is a relatively recently described phase. It is characterized by symptoms that occur after the throbbing acute headache has subsided somewhat. Patients describe this phase as a period of reduced pain but with a characteristic feeling of weakness, hangover, depressed mood, and a desire for rest.^25^ The postdrome phase is often overlooked by patients. However, in this phase, symptoms such as mood changes, difficulty concentrating, decreased appetite, muscle weakness, and fatigue may be observed.^26^ The difficulty in understanding the postdrome phase is that patients often fail to recognize that these symptoms are related to migraine. In order to understand the relationship between the postdrome phase and migraine, there are studies investigating symptom similarity between the postdrome and premonitor phases. These investigations may shed light on the mechanism of pain onset.^27^

## 2 Pathophysiology of Migraine

From the past to the present, significant progress has been made in elucidating the pathophysiology of migraine. Nevertheless, a definite pathological condition or physiological disorder that causes migraine has not been identified.^28^ It was suggested by Peter Wallwork Latham that migraine is a vascular disease.^29^ In 1938, Harold Wolf proposed the hypothesis that migraine is a disease caused by a vascular disorder.^30^ In the 1940s, ergotamine-based treatments were approved after migraine was associated with vasodilatation by Wolf.^31^ Vasodilatation of blood vessels occurs during a migraine attack. Harold Wolf stated that dilatation of vasoconstricted extracerebral blood vessels relieves pain.^32^ Vasodilatation causes inflammation of the nerves and irritation of the nociceptic nerves.^33^ Afterward, researchers thought that migraine could be a chronic neurological disorder. In 1959, the Italian neurologist Federigo Sicuteri introduced the serotonin-related medication methysergide to the therapy, but it was withdrawn due to its side effects.^34^ The main purpose of serotonin supplementation was to utilize its vasoconstrictor effect.^35^ Subsequently, β-blockers, angiotensin receptor blockers, tricyclic antidepressants, selective serotonin reuptake inhibitors, and calcium-channel blockers were used as prophylactic treatment for migraine. In addition, minerals such as magnesium, botulinum toxin, and herbal agents have been used.^36^ In 1984, Moskowitz explained the pathogenesis of migraine within the framework of the trigeminovascular system.^37^ It was hypothesized that the trigeminovascular system, during migraine, innervates the sensory trigeminal nerve fibers, the cerebral blood vessels, and the dura mater as the cause of the headache. The trigeminovascular system is the anatomical and physiological target for the treatment of migraine pain.^38^ Afferent fibers of trigeminovascular neurons innervate the meninges and surrounding vessels. Activation of these neurons releases vasoactive peptides.^39^

Serotonin is thought to be involved in the pathophysiology of migraine, and it is believed that migraine is a chronic disease caused by low brain serotonin levels.^40^ When the levels of plasma and urinary serotonin and its major metabolite 5-hydroxyindole acetic acid in patients with migraine were studied, it was observed that plasma serotonin levels were low. However, the serotonin levels in the plasma do not reflect the serotonin levels in the brain.^41^ In 1991, sumatriptan was introduced by Humphrey and colleagues as a first-line treatment in combination with NSAIDs such as ibuprofen.^42^ Other research on the pathophysiology of migraine considered increases of the neuropeptide calcitonin gene (CGRP) level in the blood.^43^ CGRP is a peptide that has receptors throughout the trigeminovascular system and whose circulating level increases during migraine attacks, as seen in clinical research.^26^ While the level of CGRP increases in the headache phase, stimulation occurs throughout the trigeminovascular system as a result of the increase in inflammatory mediators such as substance P and vasoinhibitory peptide.^2,30^ With the discovery of CGRP receptors, migraine treatment has been taken to a new dimension.^44^ Elevated CGRP levels were detected in blood samples taken during migraine attacks for the first time in 1990 by Goadsby et al., and various studies conducted in the ongoing research since then have shown the importance of CGRP in migraine.^45^ Studies with CGRP have shown that this peptide is a potent vasodilator in cerebral arteries and arterioles by activating adenylate cyclase in smooth muscle cells. CGRP is released by trigeminal nerves in response to local, cerebral vasoconstriction to dilate and maintain cerebral blood flow. These are the findings that explain the role of CGRP in the pathophysiology of migraine.^46,47^ During a migraine attack, the aim is to reduce the increased CGRP level and prevent vasodilatation caused by nitric oxide release. The main purpose of serotonin supplementation is to lower CGRP levels and inhibit vasodilatation in peripheral pathways.^35^

Opening of ion channels in meningeal blood vessels increases the sensitization of pain perception. The hypothalamus is thought to modulate these brain structures.^48^ Long-term stimulation of the trigeminovascular system and hyperexcitability of trigeminal neurons cause the nociceptive pain threshold to decrease and the pain to turn into chronic headache.^43^ Pituitary adenylate cyclase-activating polypeptide (PACAP) and pituitary adenylate cyclase-activating polypeptide type 1 (PAC1) receptors are potential targets that are expressed in the trigeminovascular system and may cause migraine attacks. Therefore, PACAP ligands may be promising for the treatment of migraine.^49^

Orexin (hypocretin) is a signaling peptide found in the hypothalamus and is thought to be associated with migraine. The orexin receptors, including orexin 1 and orexin 2, can manage neurotransmission in the trigeminovascular systems in animal experiments, but clinical trials with dual orexin receptor antagonists have not been successful.^50^ It has been suggested that glutamate may be potentially effective in the treatment of migraine due to its important role in cortical stimulation in the brain. However, no effective way has yet been found to use it in the acute and preventive treatments of migraine. The fact that it is one of the main receptors of the nervous system makes it difficult to treat migraine without causing side effects. In the future, migraine-related disordered glutamergic signaling may be targeted without altering normal brain function.^51^ Transient receptor potential (TRP) channels and acid-sensing ion channels (ASICs) could be potential targets for migraine therapy.^52^ Due to the role of TRP channels in trigeminal nociception, some compounds have been clinically evaluated as TRP antagonists for the treatment of migraine in recent years. Also, ASICs have a role in cortical spreading depression in the brain. Currently, studies are being carried out on ASIC antagonists, considering that they may be the underlying cause of migraine with aura.^49^

**Figure 2:**
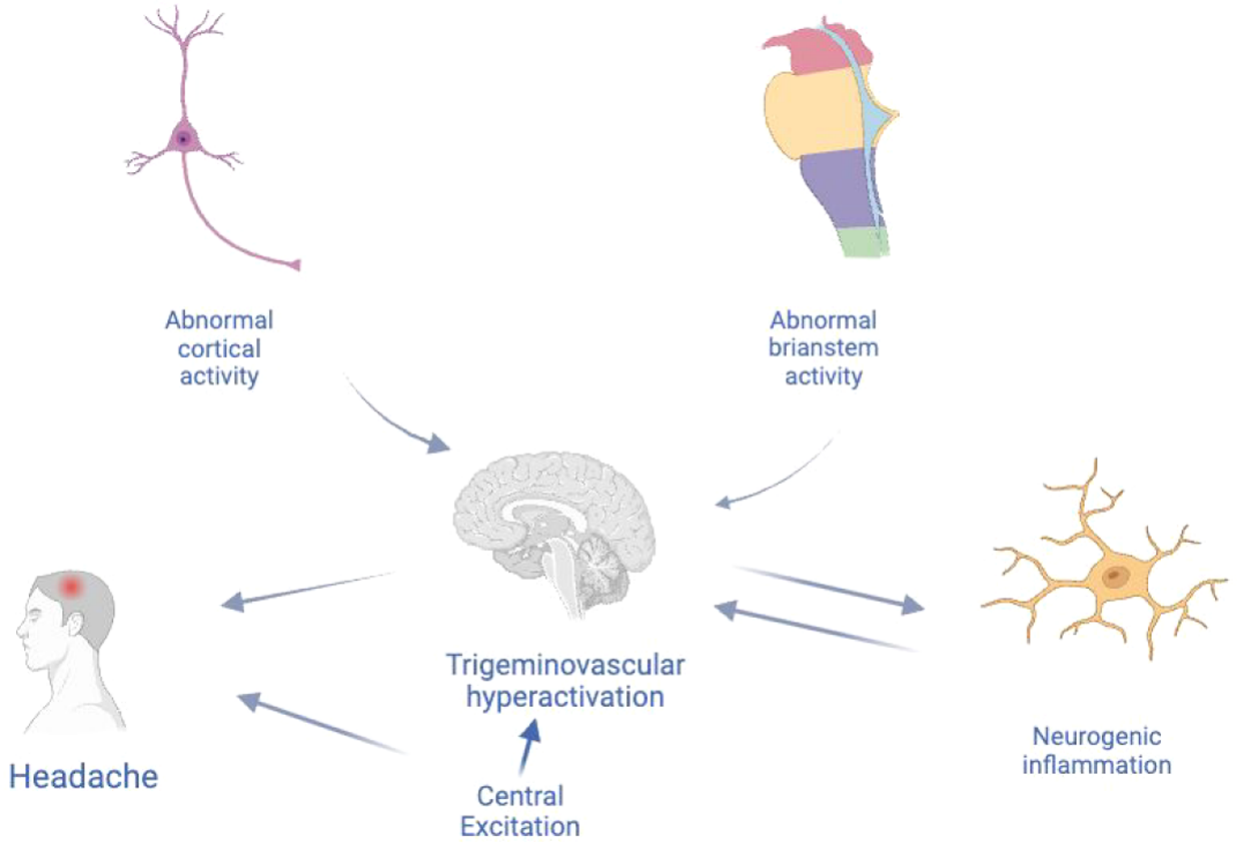
Migraine mechanism summary.

In the light of this information, we look at migraine from two different perspectives: peripheral and central. Peripheral signals from sensory stimuli initiate a migraine attack. Central nervous system (CNS) dysfunction is the most important event triggering migraine.^13^ One CNS-related idea is that neurological symptoms occur as a result of central disorders and environmental stimuli causing psychological stress.^53^ CNS excitation and inhibition imbalance are the causes of migraine.^46^ Three different complex chains of events are believed to be the causes of migraine: nervous system (trigeminal system), immune system (satellite cells), and vascular system (intracranial dual arteries)^54,55^ (Figure 2).

## 3 Diagnosis of Migraine

Migraine is classified into two major clinical categories, with and without aura. These classes differ from each other in terms of clinical features and biological susceptibility factors.^54^ The responses of these two clinical classes to drugs and the effectiveness of drugs on each clinical class are different.^56^ While the prevalence of migraine is 10% in the world, 70% of cases are without aura.^46^ The diagnosis of migraine is made by evaluating the slowly developing headache and associated symptoms. The International Headache Classification Code is 1.2 for migraine with aura and 1.1 for migraine without aura. The symptoms occurring in migraine with aura can be confused with transient ischemic attack or occipital epilepsy, but the diagnosis can be made by considering the onset of the symptoms, the style, the visual symptoms, the duration, and the age of the patient.^57^ Migraine with aura is caused by intracerebral vasoconstriction, while migraine without aura is a neurobiological disorder^53^ (Table 1).

**Table 1:** Classification of Migraine

1.2 Migraine with aura	1.1 Migraine without aura
• visual, sensory, speech	• the period of migraine can vary between 4 and 72 h
• headache begins with aura	• intense headache on one side
• may be confused with sinusitis or normal headache when determining the diagnosis	• progressively increasing headache daily routine
• may be confused with ischemic attack or occipital epilepsy	• accompanied by gastrointestinal symptoms
• intracerebral arterial vasoconstrictions	• neurobiological disorder

## 4 Migraine and Other Diseases

### 4.1 Cardiovascular Diseases

Studies have shown that there may be a relationship between cardiovascular disease and migraine disorders. The likelihood of migraine with aura may increase in individuals with cardiovascular disease, especially in those who have had ischemic stroke. In addition, patients with migraines are more likely to have cardiovascular diseases such as atrial fibrillation and myocardial infarction. It is possible that there is a relationship between migraine medications and cardiovascular diseases. The mechanism between them is not known with certainty, and definitive evidence is needed to understand it. Vascular conditions in migraine increase the comorbidity of cardiovascular diseases.^58,59^ Medications prescribed for acute and prophylactic treatment of migraine patients may have cardiovascular side effects. Therefore, new drug research should focus on minimizing cardiovascular side effects along with reducing all possible side effects.

### 4.2 Gastrointestinal Diseases

There is a statistical correlation between GI diseases and migraine disease. Migraine patients have been reported to have a higher incidence of GI disorders than healthy people. In addition, the effectiveness of migraine medications used in patients with GI system diseases is likely to be decreased. This may adversely affect the effectiveness of migraine medications. During acute migraine attacks, effects include decreased GI motility and impaired drug absorption. Also some GI symptoms including nausea may adversely affect migraine treatment. When the mechanisms of both diseases are fully elucidated, then the pathophysiological relationship between them will be clearly understood.^60^

### 4.3 Multiple Sclerosis

It is known that migraine patients have a higher risk of developing MS than healthy individuals. Migraine symptoms are known to increase comorbidity in patients with MS. However, early diagnosis by neurologists and appropriate management of the disease can reduce this risk factor. MS and migraine are both more common in women, and epidemiological similarities have been observed. It has also been observed that migraine patients have a higher risk of developing MS. Some of the known connections between MS and migraine are a temporal relationship and high sensitivity to C-reactive protein levels. Despite all these associations, the relationship between migraine and MS has not yet been fully resolved.^61,62^

### 4.4 Epilepsy and Migraine

Migraine and epilepsy are distinct neurological disorders with specifically different clinical features, but despite this, clinical diagnosis can be difficult due to the similarity of symptoms. The pathophysiological mechanism of headache triggered by seizures is being investigated. Throbbing headache may cause migraine seizures.^63^ Epidemiologic studies have revealed the association of migraine with epilepsy. Both are diseases with proven comorbidity. MS and migraine are both disorders related to transmembrane permeability. The use of common drugs in the treatment of both diseases also supports the relationship between them. Despite all these findings, questions remain.^64^

### 4.5 Anxiety

According to studies conducted to understand the relationship between migraine and anxiety, it was observed that the anxiety factor was higher in patients with migraine than in those without migraine. In addition, anxiety is more common in patients with chronic migraine than in patients with acute migraine. Migraine and anxiety can lead to diseases such as hypertension and obesity.^65^ As a result of studies, it has been discovered that there is a multifaceted relationship and common mechanisms between the mechanisms of migraine and depression.^66^

## 5 Treatment of Migraine

The main goal of migraine treatment is to reduce the severity of pain during an attack and to prevent migraine. Prophylactic treatment is necessary for chronic migraine patients, whereas in acute migraine patients, treatment may be symptom-oriented due to the low frequency of attacks.^13^ Two types of treatment have been developed for migraine: acute treatment, which terminates the attack, and preventive treatment, which is recommended to reduce the frequency and severity of attacks. When the two treatments are compared, acute therapies offer the patient a quick and complete recovery with the fewest side effects.^56^ Due to their poor efficacy, side effects, and low tolerability, the drugs used in preventive treatment for migraine are limited.^67^ In addition to these treatment methods, non-pharmacological treatments are also applied.

### 5.1 Acute Treatment

The goal in the acute treatment of migraine is the relief of headache and migraine-related symptoms within two hours. Compounds used for this purpose can be classified as analgesics, NSAIDs, triptans, gepants, ditans, and ergot alkaloids. The migraine characteristics of the patient and the failures of previous treatments are determinants in the choice of medication for treatment.^68^

### 5.2 Preventive Treatment

Preventive treatment aims to reduce the frequency and severity of attacks in patients who usually have migraine attacks more than two days a month. Drugs used in preventive treatment for migraine are generally antihypertensives, antidepressants (amitriptyline), anticonvulsant agents (topiramate, sodium valproate), and calcium channel blockers (flunarizine). The efficacy of topiramate and onabotilinumtoxin A (botox) has been proven in the treatment of chronic migraine.^69,70^

### 5.3 Non-pharmacological Treatments

Non-pharmacological treatments are known as stand-alone or adjunctive treatments to pharmacological drugs. Treatments such as cognitive behavioral therapy, biological feedback, and relaxation training are applied as non-pharmacological treatment approaches with the highest effectiveness.^71^ There are also methods that are less effective, such as physical therapy, sleep management, acupuncture, and diet regulation. It can be said that non-pharmacological treatments minimize drug use with a multidisciplinary approach in addition to clinical methods.^3^

## 6 Migraine Drugs and Their Synthesis

There are many drugs for the acute or preventive treatment of migraine that have already been approved by the FDA for other diseases. With the discovery of the mechanism of migraine over time, drugs that can be used in its treatment have been approved by the FDA. Ergot alkaloids,^72^ valproate, topiramate,^73^ β-blockers,^74^ calcium channel blockers,^75^ and amitriptyline^76^ are the most commonly used drugs in migraine treatment.

### 6.1 Analgesics

Paracetamol and NSAIDs (ibuprofen, aspirin, diclofenac, naproxen) are mostly preferred as the first choice in the treatment of acute migraine because of their analgesic effects, while the use of paracetamol alone is not preferred. If these drugs fail, triptans are used. Among these compounds, the least effective compound is naproxen, which requires high doses.^77^ The use of analgesics in the treatment of migraine was approved by the FDA some time after they were approved for other diseases.

### 6.2 Triptans

Triptans are migraine-specific drugs. Although they are used for moderate or severe headache, which is one of the most severe symptoms of migraine, they are recommended to be taken during mild headache, which is the first phase of a migraine attack. The superiority of all triptans over placebo has been clinically proven. The mechanism of action of triptans is based on increasing the serotonin signal by stimulating the serotonin receptors in the cranial blood vessels and nerve endings and inhibiting the release of peptides such as CGRP and substance P. Triptans provide high treatment success by selectively binding to 5-HT1B/1D receptors. It is thought that they may have cardiovascular side effects due to their vasoconstrictor effects on smooth muscles.^2,78^ Structure–activity studies on triptan and its derivatives have shown that substituents at the 5-position increase the agonist activity of the 5-HT1B receptor, and the tryptamine moiety provides hydrophobic, ionic, and hydrogen bond interactions.^79^

#### Sumatriptan

**Figure 3:**
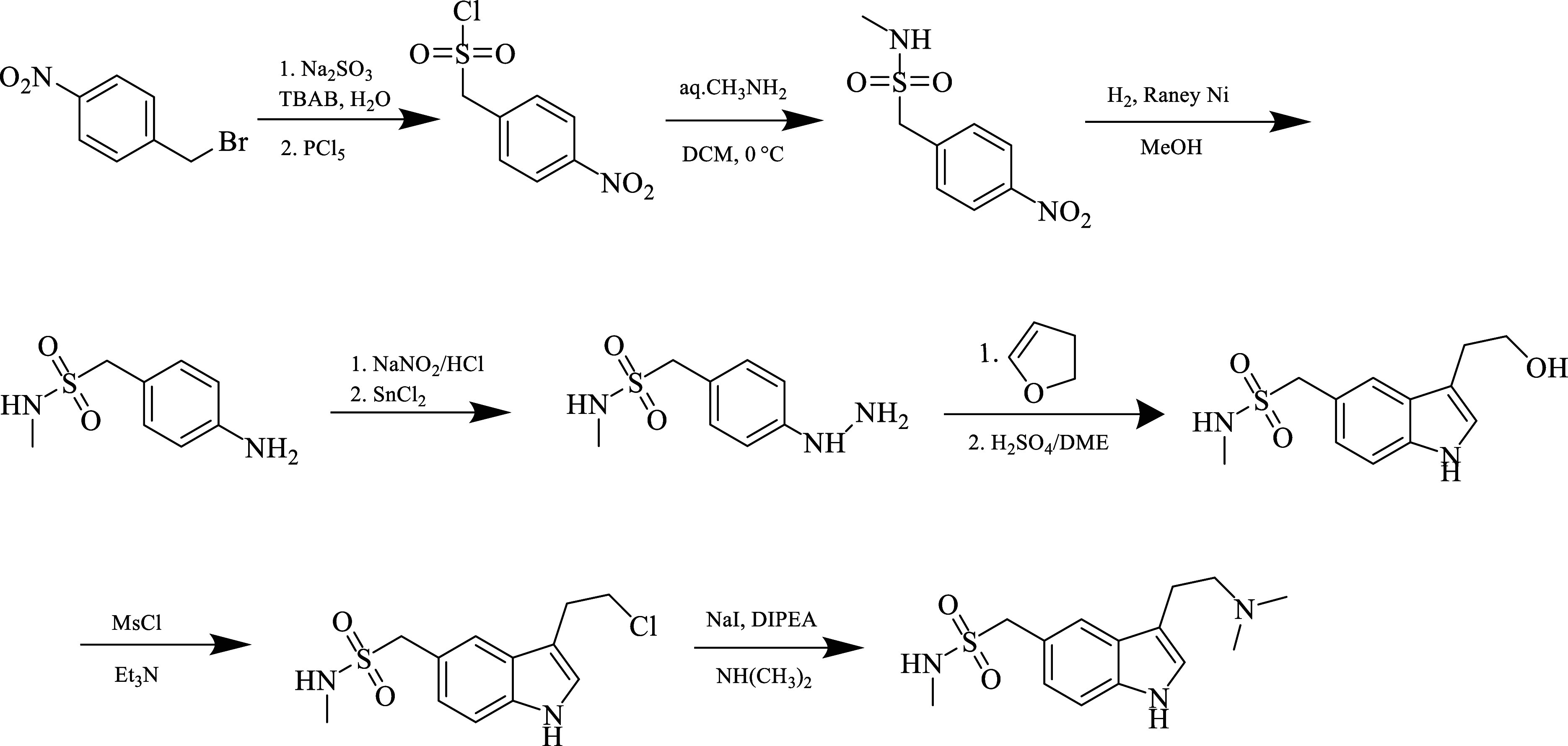
Synthesis of sumatriptan.

Sumatriptan (1-[3-[2-(dimethylamino)ethyl]-1*H*-indol-5-yl]methylmethanesulfonamide) was the first approved triptan compound. Its mechanism of action is related to its vasoconstriction as a 5-HT1B/1D agonist. In addition, in 2019, 5-HTF receptor agonist activity was proven by the FDA in acute migraine attacks. Some cases of inflammation reduce the level of cytokines without causing any significant side effects. There are combined preparations with the COX1/2 inhibitor naproxen.^80^ In children over 12 years of age and adolescents, the usefulness of sumatriptan/naproxen sodium has been proven. It has poor pharmacokinetics due to its low bioavailability and short half-life. Sumatriptan is metabolized mainly by MAO-A to inactive indole acetic acid and glucuronide conjugate, whereas naproxen undergoes hepatic microsomal oxidation. Use in the form of tablets once a day reduces unnecessary drug use.^81^ Its synthesis is shown in Figure 3.

The reaction of 4-(nitrophenyl)methanesulfonyl chloride from 1-(bromomethyl)-4-nitrobenzene with methylamine gives the appropriate sulfonamide derivative. It is converted to -NH_2_-, which is obtained by catalytic reduction of the nitro group in the compound, and turns into an aryl hydrazine intermediate with NaNO_2_ and SnCl_2_. Reaction of this intermediate with dihydrofuran gives the indolyl alcohol derivative. Following the conversion of the alcohol structure to chlorine, amination with dimethyl amine gives sumatriptan.^82^

#### Zolmitriptan

**Figure 4:**
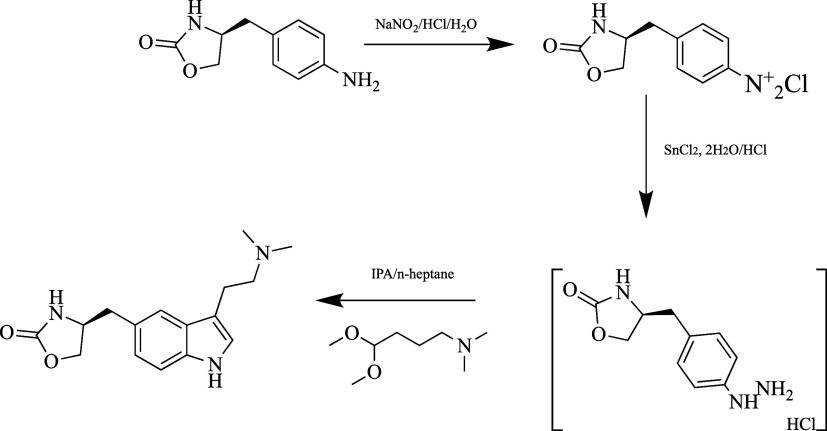
Synthesis of zolmitriptan.

Zolmitriptan ((*S*)-4-[3-[2-(dimethylamino)ethyl]-1*H*-indole-5-methyl]-2-oxazolidone) is a non-selective 5-HT1B/1D receptor agonist triptan derivative approved by the FDA in 1997. Both 2.5 and 5 mg doses are available in tablet form. It can be taken up to a maximum dose of 15 mg per day. It has been developed for the treatment of acute migraine. Its formulation as a nasal spray allows its use in children. Zolmitriptan is a drug of choice in the treatment of migraine because of its high oral bioavailability, lipophilic character, and active hepatic metabolite.^83^ The 3-methylindole ring carried by the compound is responsible for the toxicity of zolmitriptan.^84^ Its synthesis is shown in Figure 4.

Reduction of the diazonium salt formed as a result of diazotization of 5-[4-(4-aminobenzyl)]oxazolidin-2-one with sodium nitrite/hydrochloric acid with tin chloride gives the intermediate product with the hydrazine structure. Zolmitriptan is obtained by the reaction of this intermediate with 4,4-dimethoxy-*N,N*-dimethylbutan-1-amine.^85^

**Figure 5:**
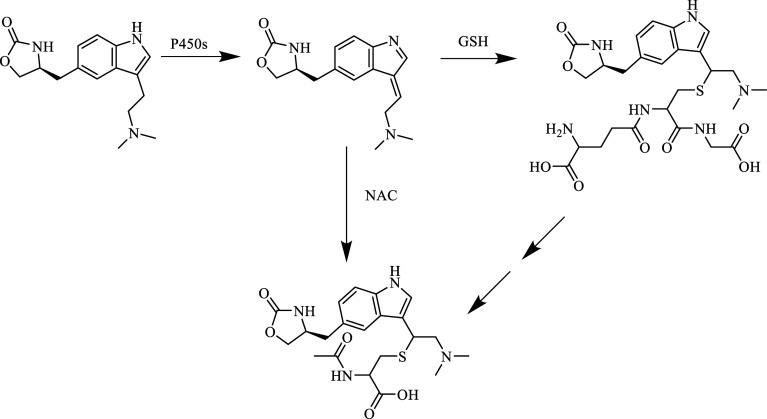
Suggested biotransformation pathways for zolmitriptan.

Metabolism studies of zolmitriptan have shown that it is N-demethylated by CYP1A2. The resulting metabolite is converted to indole ethyl alcohol by MAO-A and excreted as the indole acetic acid metabolite. Indole acetic acid is the most abundant metabolite in humans. In addition, in a study conducted on mice, it was observed that the CYP2D6 enzyme was the predominant gene in the activation of zolmitriptan. According to predictions, bioactivation is achieved by oxidation of the imine in the indole group. Subsequently, GSH conjugation takes place.^84^ In a study by Yu et al., it was reported that zolmitriptan was an inducer of CYP3A2 in male rats.^86^ The possible metabolism pathways for zolmitriptan are shown in Figure 5.

#### Naratriptan

Naratriptan (*N*-methyl-2-[3-(1-methylpiperidin-4-yl)-1*H*-indol-5-yl]ethanesulfonamide) is a widely used non-selective 5-HT1B/1D receptor agonist approved by the FDA in 1998. As a result of clinical studies, naratriptan is known to reduce mild headache at doses of 2.5 mg. An oral tablet taken 3–4 times a day is equivalent to a 100 mg dose of sumatriptan. In addition, the cardiovascular side effects of naratriptan are very low.^87^ While 70% of naratriptan is excreted unchanged, a significant portion is metabolized by cytochrome P450 enzymes. It has the longest half-life among triptans. With this feature, recurrence of headache is less common.^88^

**Figure 6:**
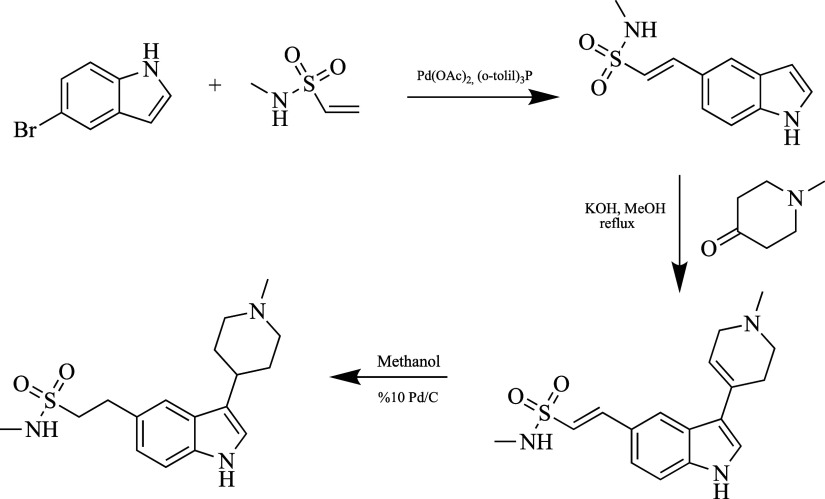
Synthesis of naratriptan.

There are many different routes for the synthesis of naratriptan, one of which is the synthesis by Oxford University, shown in Figure 6: starting with 5-bromoindole, Pd(OAc)_2_-catalyzed reaction with *N*-methyl vinyl sulfonamide gave the molecule that reacted with *N*-methyl-4-piperidone to form an intermediate compound in the presence of KOH and methanol. Naratriptan was obtained by reduction of the intermediate compound.^89^

#### Rizatriptan

Rizatriptan (2-(5-((1*H*-1,2,4-triazol-1-yl)methyl)-1*H*-indol-3-yl)-*N,N*-dimethylethanamine) is a widely used non-selective 5-HT1B/1D receptor agonist approved by the FDA in 1998.^90^ Rizatriptan is a second-generation triptan. While its rapid action makes it superior to other triptans, its metabolism in the liver reduces its oral bioavailability to 45%. It causes side effects on the cardiovascular, gastrointestinal, and respiratory systems. For this reason, a low oral dose of 5–10 mg is recommended.^91^

**Figure 7:**
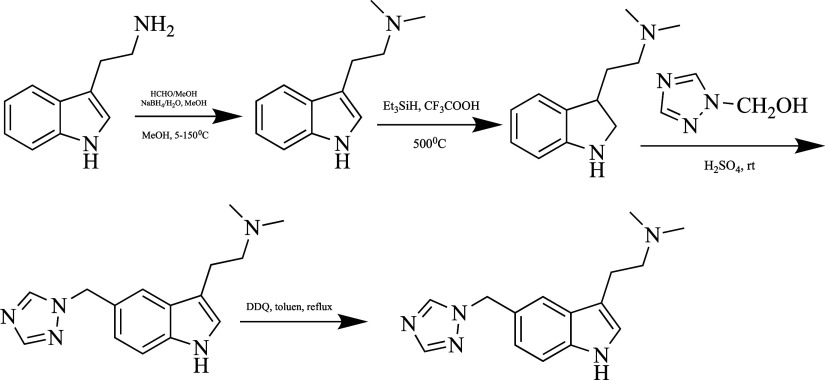
Synthesis of rizatriptan.

Studies have shown that rizatriptan has more than one synthesis method. Mostly, an intermediate product containing an indole ring is formed by the Fischer indole reaction.^92^ Rizatriptan is synthesized in multiple steps under the conditions shown in Figure 7, starting from tryptamine. This new method is industrial. Studies on its use are still ongoing.^90^

#### Almotriptan

**Figure 8:**
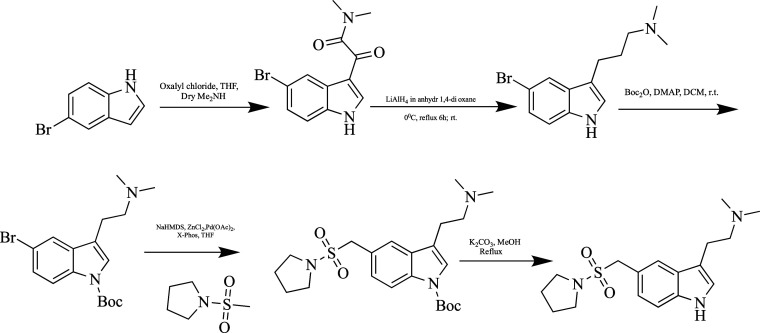
Synthesis of almotriptan.

Almotriptan (*N,N*-dimethyl-2-[5-(pyrrolidin-1-ylsulfonylmethyl)-1*H*-indol-3-yl]ethanamine) is a widely used non-selective 5-HT1B/1D receptor agonist approved by the FDA in 2001. Almotriptan is the first triptan approved by the FDA for the treatment of headache with or without aura lasting more than 4 h in adolescents. It is a safe triptan for use in migraine and other types of headache, especially in the pediatric population.^93^ Almotriptan is approved for use in monotherapy and in combination with NSAIDs.^56^ The most common known side effects are dizziness, drowsiness, and fatigue. While as much as 50% of almotriptan is excreted in the urine, the remaining amount is oxidized via CYP3A4 and CYP2D6 to inactive metabolites. Its oral bioavailability is 70%, and it is well tolerated by the body and has a low side effect profile.^94^ Its synthesis is shown in Figure 8.

An impure compound was obtained with 5-bromo-1*H*-indole, oxalyl chloride in anhydrous tetrahydrofuran (THF), and dry Me_2_NH gas. The formed intermediate was reduced with LiAlH_4_ and protected by the *tert*-butoxycarbonyl (Boc) group in the next step. The resulting molecule was combined with 1-(methylsulfonyl)pyrrolidine under Negishi conditions. Finally, a clean and purifiable almotriptan molecule was obtained by getting rid of the Boc group with a mixture of K_2_CO_3_ in methanol solvent.^95^

#### Frovatriptan

Frovatriptan ((6*R*)-6-(methylamino)-6,7,8,9-tetrahydro-5*H*-carbazole-3-carboxamide) is a widely used non-selective 5-HT1B/1D receptor agonist approved by the FDA in 2001. Its moderate affinity for 5HT7 receptor in in vitro studies also contributes to its pharmacological properties. It has 4 times more affinity for 5-HT1B receptor than sumatriptan. Its oral bioavailability is between 22 and 30%.^96^ Its half-life is 26 h, which is longer than those of other triptans. Since it is eliminated by both the kidneys and liver, it is advantageous for migraine patients who have a disorder in one of them. The lack of interaction with other drugs is evidence of its superiority over other triptans. It is metabolized by monoaminoxidase or CYP3A4 enzyme. Frovatriptan profiles at low doses are similar to those of other triptans. Between 2.5 and 40 mg, the dose–response curve is flat compared to other triptans. Up to 10 mg, there is no significant increase in side effects. Even the side effects above 10 mg are mild.^97^ It has cardiovascular side effects, although less than other triptans. There are studies proving the efficacy of frovatriptan in premenstural migraine and its acute treatment.^98^

**Figure 9:**
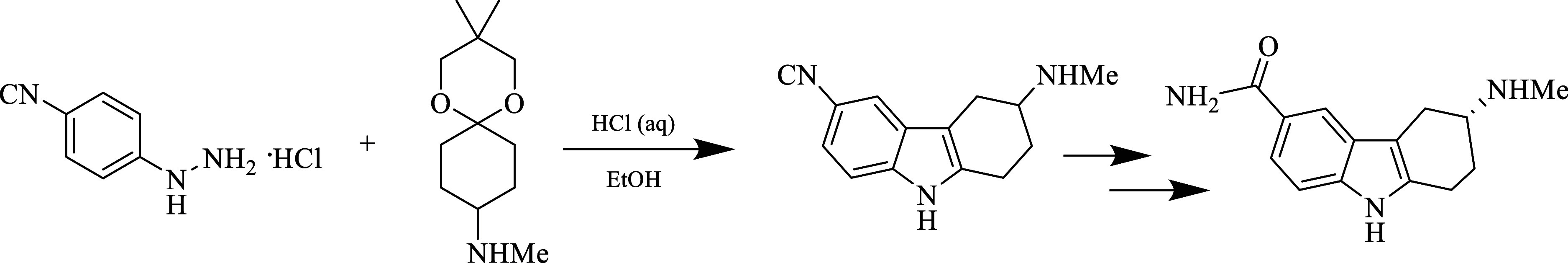
Synthesis of frovatriptan.

The tetrahydrocarbazole derivative compound frovatriptan is synthesized according to the Fischer indole method (Figure 9) which is frequently used in industry.^99^

#### Eletriptan

**Figure 10:**
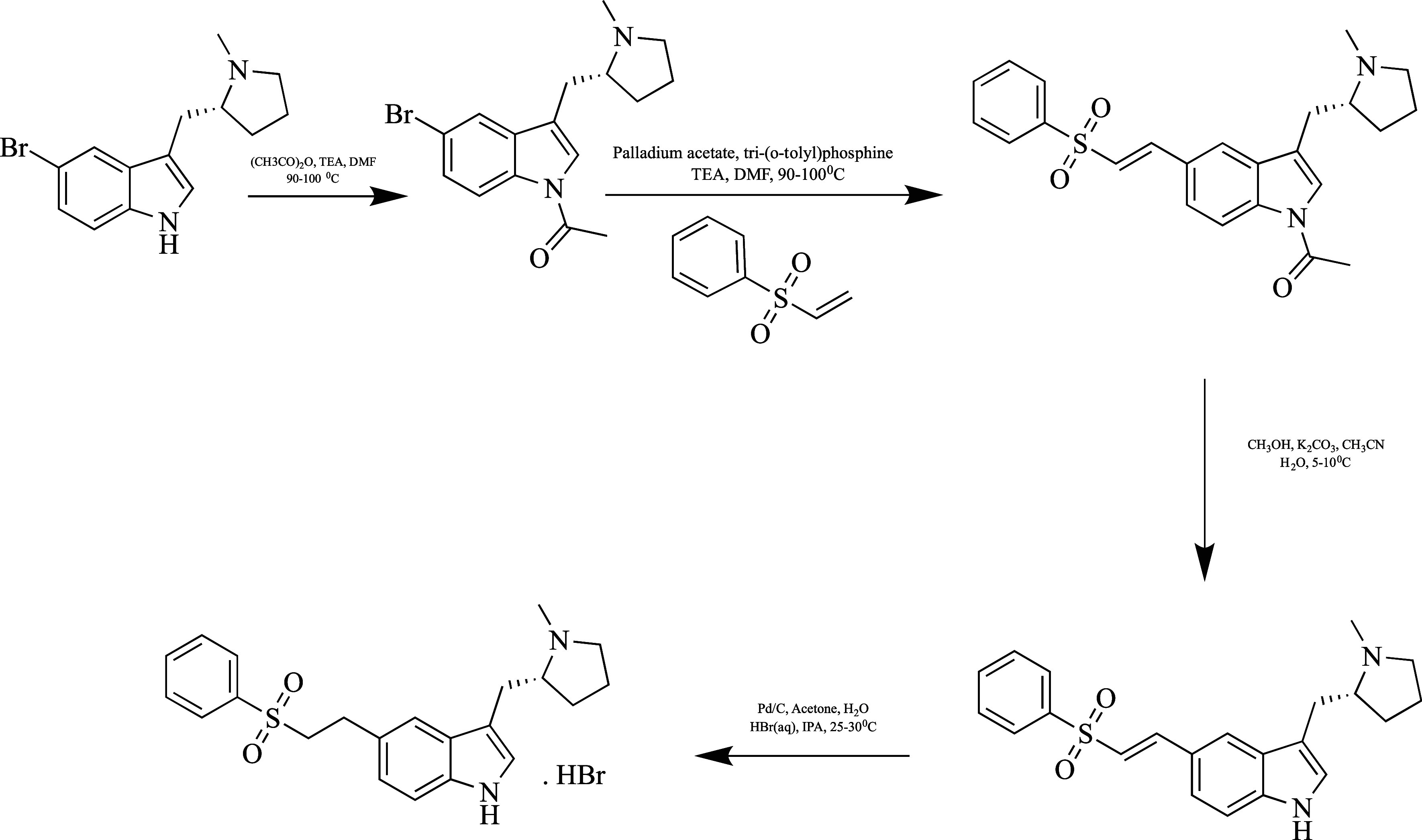
Synthesis of eletriptan.

Eletriptan (5-[2-(benzenesulfonyl)ethyl]-3-[[(2*R*)-1-methylpyrrolidin-2-yl]methyl]-1*H*-indole) is a widely used non-selective 5-HT1B/1D/1F receptor agonist approved by the FDA in 2002. The clinical dose of eletriptan is 40 mg. Eletriptan is a triptan with high clinical efficacy. It is particularly well tolerated in individuals without coronary disease due to the cardiovascular side effects of triptans. It is metabolized by the CYP3A4 enzyme. It is more efficient than other triptans. Especially if it is prescribed together with serotonin derivative drugs which are strong CYP3A4 inhibitors, the evaluation of the disease should be done well. Bioavailability is high and reaches up to 50%.^100,101^ Its synthesis is shown in Figure 10.

The compound obtained by acetylation of (*R*)-5-bromo-3-(*N*-methylpyrrolidin-2-yl-methyl)-1*H*-indole under certain conditions was combined with phenyl vinyl sulfone under Heck reaction conditions. Deacetylation of the resulting compound was achieved, and eletriptan hydrobromide was formed upon treatment with hydrobromic acid.^102^

#### Ditan Derivative: Lasmiditan

**Figure 11:**
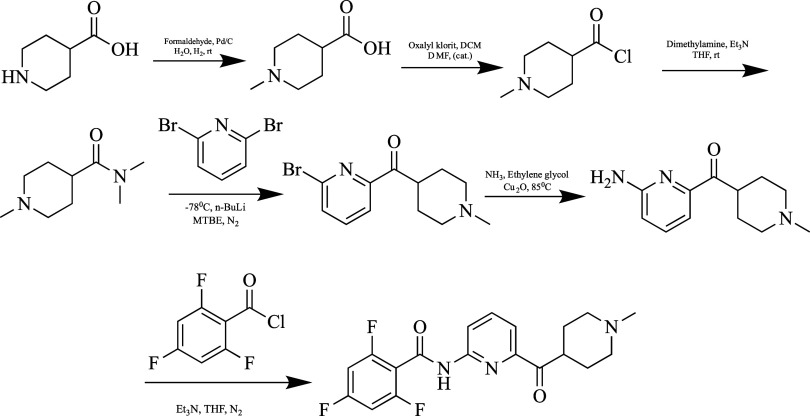
Synthesis of lasmiditan.

Today, lasmiditan is the only ditan available.^103^ The 5-HT1F receptor agonist lasmiditan is a centrally acting, selective, and high-affinity compound. It acts on the trigeminovascular system, inhibiting neurotransmitter release without causing vasoconstriction.^104^ It was approved by the FDA in 2019; the FDA recommends a dose of 50–100 mg. The dose should not exceed 200 mg per day. It has a bioavailability of approximately 40%. It starts to show its effect 30 min after taking it. It is similar to triptans but has milder side effects than them.^105^ Its synthesis is shown in Figure 11.

First, the 1-methylpiperidine-4-carboxylic acid molecule was obtained according to Borch reduction conditions. After the carboxylic acid group was chlorinated, a coupling reaction with the 2,6-dibromopyridine molecule took place in two steps. Finally, after amination of the formed molecule, it was combined with acyl chloride and lasimiditan was obtained.^106^

### 6.3 CGRP-Dependent Therapies

CGRP is a 37 amino acid peptide. It functions as a potent vasodilator in all vascular tissues. CGRP is effective on the trigeminovascular system, which has an important role in the pathophysiology of migraine. In patients with migraine, the level of CGRP in the blood increases during a chronic migraine attack. The CGRP level in the blood can be accepted as a biomarker for chronic migraine.^107^

Monoclonal antibodies and small-molecule drugs are used in CGRP-receptor-dependent therapies. Since CGRP and the CGRP receptor are located in the peripheral and central trigeminovascular system pathway, they mediate vasodilation and pain signaling along this pathway. Based on this, monoclonal antibodies targeting the CGRP pathway have been offered for treatment. However, difficulties that reduce patient compliance, such as monthly or quarterly parenteral administration of these drugs, have compelled the development of new, alternative CGRP antagonists. Due to the hepatotoxicity seen in the first-generation drugs, the second- and third-generation oral CGRP receptor antagonists aimed to eliminate these harmful effects.^108^ In new CGRP-dependent treatment methods, direct blockade of CGRP or its receptor has been studied.^109^ In the past 25 years, antagonism of the CGRP pathway has provided great success for individuals with migraine. These treatment modalities have proven to be important for the acute and prophylactic treatment of migraine.^110^ Small-molecule CGRP receptor antagonists are effective in the acute treatment of migraine, while monoclonal antibodies are useful in the preventive treatment of chronic migraine.^111^

#### 6.3.1 Anti-CGRP Monoclonal Antibodies

Due to the side effects of drugs used in the treatment of migraine, there is always a need to investigate new classes of drugs. In 2018, monoclonal antibodies (mAbs) took their place in the clinic as a treatment targeting the CGRP receptor. This class of drugs are large-molecule drugs used in migraine prophylaxis. Erenumab is an mAb against the CGRP receptor, while eptinezumab, fremanezumab, and galcanezumab bind to the CGRP receptor. Constipation, swelling at the injection site, upper respiratory tract infection, nausea, and pain are common side effects of mAbs.^112^

##### Erenumab (AMG334)

Erenumab, known by its market name as Aimovig, is an mAb used in the prophylactic treatment of migraine, approved by the FDA in 2018 against the CGRP receptor and CGRP ligand. The crystal structure of the complex formed with CGRP provides the direct ligand blocking mechanism.^113^ According to clinical studies, erenumab binds 5000 times more selectively and with higher affinity than other CGRP-dependent treatments.^114^ The recommended monthly dose of erenumab is 70 mg, and there are data showing that clinical benefits can be seen at a dose of 140 mg.^115^ Because of the short half-life of mAbs, daily intake may be required compared to CGRP receptor antagonists.^116^

##### Galcanezumab (LY2951742)

Galcanezumab is the first mAb to potently and selectively block the CGRP receptor without blocking its biological activity.^117^ It is used in the preventive treatment of migraine and to reduce the frequency of headache attacks. Phase 2 and Phase 3 studies have shown it to reduce the number of migraine headache days per month. These studies have demonstrated that galcanezumab is a safe treatment.^118^ After a 240 mg loading dose, it can be administered subcutaneously as a 120 mg/month dose. The most common side effects reported by patients include nasopharyngitis and pain at the injection site.^119^

##### Fremanezumab (TEV-48125)

Fremanezumab is a mAb approved by the FDA in 2018. The recommended dose is 225 mg subcutaneously every month or 675 mg every 3 months. It should be injected into the upper arm or abdomen.^120^ Currently approved for use in adults, it has shown significant results in the preventive treatment of chronic and episodic migraine in clinical trials.^121^ It was observed that the use of fremanezumab every 3 months or once a month resulted in improvement in migraine headache for up to 12 months.^122^ More studies are needed to explore its long-term effects.

##### Eptinezumab (ALD403)

Approved by the FDA in February 2020, the clinically recommended dose of eptinezumab is 100 mg as an intravenous infusion every 3 months. 300 mg may be used in some patients. Although preclinical and clinical studies are promising, there is still not enough information about its long-term pharmacokinetics and metabolism.^123^ It specifically binds to CGRP and blocks CGRP-related pain signaling pathways. This is the only drug used as an intravenous infusion, thus offering pharmacokinetic advantages over mAbs used subcutaneously.^124^

#### 6.3.2 Gepants

With the discovery of gepants, migraine treatment has gained a new perspective. They have proven to be superior to triptans in terms of safety. They have been reported to relieve pain within 2 h and greatly reduce the frequency of migraine attacks. No significant hepatotoxic or cerebrovascular side effects were observed. Gepants are used in both acute and preventive treatments. They are used in periods preceding a headache. Rimegepant is the only gepant approved for acute treatment, and atogepant is for chronic treatment. Ubrogepant is the only one approved for use in the prodrome phase and acute attacks. Zavegepant is the only nasal gepant. Gepants are more suitable in pregnancy and offer greater ease of use compared to mAbs.^125,126^

##### Ubrogepant

**Figure 12:**
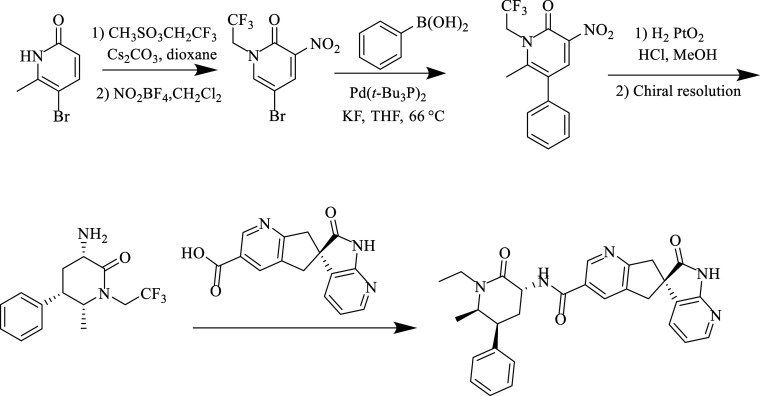
Synthesis of ubrogepant.

Ubrogepant is a CGRP receptor antagonist approved by the FDA in 2019. It is used in the treatment of acute migraine. It is the first drug approved for migraine with and without aura in adults. In the treatment of acute migraine, 50–100 mg doses are considered safe and can be administered up to a maximum dose of 200 mg. It is recommended to use a 50 mg dose in patients with renal and hepatic impairment. Ubrogepant is metabolized by CYP3A4 90 min after injection. Apart from the parent compound, two other glucuronate conjugates are approximately 6000 times more selective for the CGRP receptor, with a half-life of 5–7 h.^127^ They do not have the hepatotoxicity that the first-generation derivatives have. Ubrogepant is a highly selective gepant. Side effects of mild severity include nausea, drowsiness, and dry mouth.^128^ Its synthesis is shown in Figure 12.

After first N-alkylation and nitration of 5-bromo-6-methylpyridin-2(1*H*)-one, 1-(2,2,2-trifluoro)ethyl-3-amino-5-phenyl-6-methylpiperidin-2-one is obtained by reduction of the 3-nitro-5-phenylpyridin-2(1*H*)-one. After being separated into its chiral enantiomers, ubrogepant was obtained as a result of the formation of an amide bond with an intermediate containing carboxylic acid with a 3-aminopiperidone derivative.^129^

##### Rimegepant

**Figure 13:**
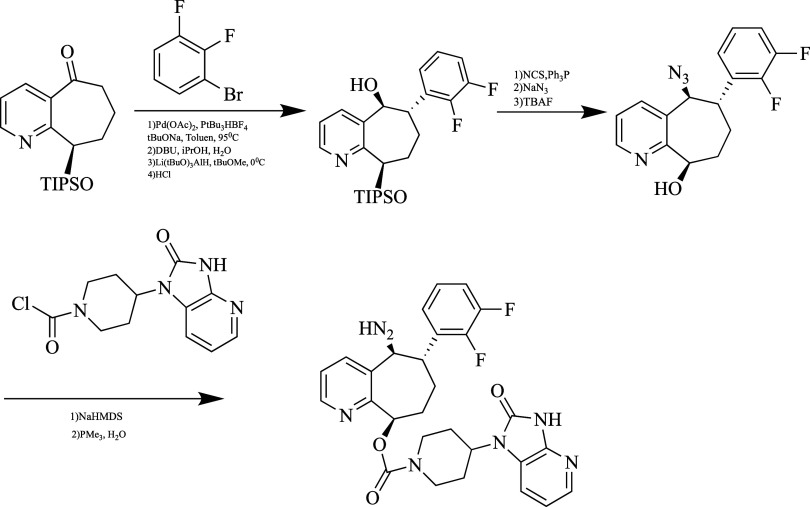
Synthesis of rimegepant.

Approved in 2020, rimegepant is the first CGRP receptor antagonist FDA-approved for acute migraine attacks and also preventive treatment. It is a new therapeutic agent developed to effectively treat migraine without the cardiovascular side effects and hepatotoxicity caused by triptans.^130^ The primary amine group carried by the cyclohepta[*b*]pyridine ring in the compound increases the water solubility and polarity while maintaining the permeability of the lipid membrane, thus providing good pharmacokinetic properties.^46^ In Phase 3 studies, the use of 75 mg in migraine attacks has proven its efficacy and safety. It has a usefulness that improves the quality of life and prevents overuse of medicines.^131^ Its synthesis is shown in Figure 13.

First, the triisopropylsilyl (TIPS) group was attached to the alcohol group to achieve stereochemical control of the 7,8-dihydro-5*H*-cyclohepta[*b*]pyridine-5,9(6*H*)-dione compound. Palladium-catalyzed α-arylation of this TIPS-protected compound and ketone reduction were performed in accordance with the literature. The resulting alcohol group was first converted to chloride to obtain a chiral center. By treating the chiral center formed as a result of this step with NaN_3_ and deprotecting the TIPS group, the (5*S*,6*S*,9*R*)-5-azido-6-(2,3-difluorophenyl)-6,7,8,9-tetrahydro-5*H*-cyclohepta[*b*]pyridin-9-ol group compound was obtained. The 4-(2-oxo-2,3-dihydro-1*H*-imidazo[4,5-*b*]pyridin-1-yl)piperidine-1-carbonyl chloride compound synthesized in 5 steps, which is the key reaction of rimegepant synthesis, was substituted to the alcohol group under certain conditions. Finally, the azide group was reduced to the amine with PMe_3_.^114,132,133^

##### Atogepant

**Figure 14:**
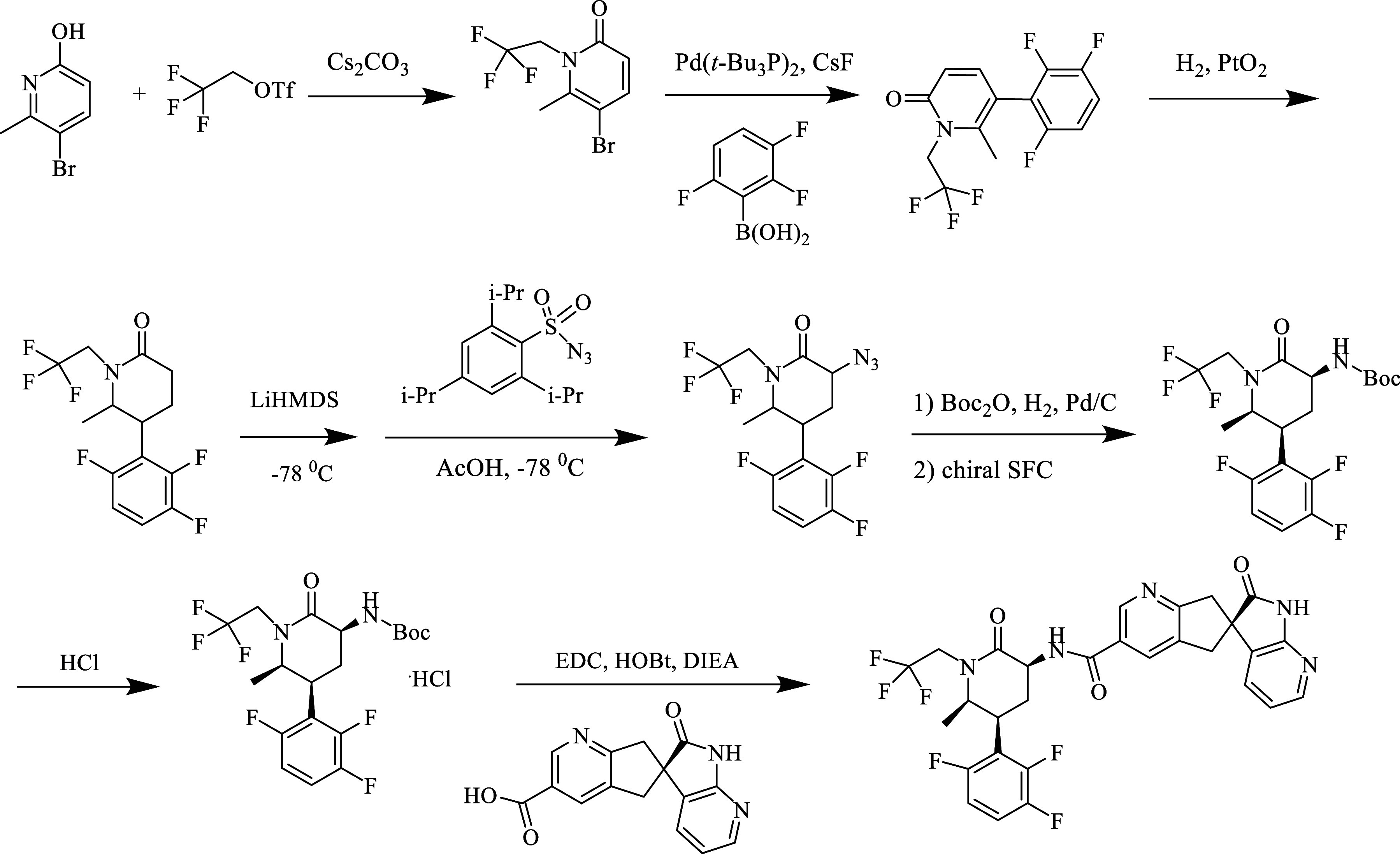
Synthesis of atogepant.

Atogepant is a CGRP receptor antagonist that does not cause hepatotoxicity and cardiovascular side effects and was approved by the FDA in 2021 for the treatment of acute migraine. A 12-week course of treatment with administration of between 10 and 120 mg is safe and tolerable. Significant side effects were not observed.^134^ Since it contains a large number of fluorine atoms, it has the property of H bond acceptor and thus has high interaction and affinity with the receptor. It reduces inflammation and pain sensitivity by blocking CGRP receptors in a non-competitive way in the prophylaxis of episodic migraine. Atogepant is a preventive agent for migraine pain.^135^ It is the first drug licensed among gepants due to its long half-life and low side effects on the heart and liver.^136^ Its synthesis is shown in Figure 14.

As a first step, N-alkylation of the 5-bromo-6-methylpyridin-2-ol ring was achieved with 2,2,2-trifluoroethyl triflate under Cs_2_CO_3_ catalysis. In the next step, the diaryl derivative was obtained under Suzuki reaction conditions. After the hydrogenation of the pyridine ring, azidation of the obtained molecule was achieved. Then, the obtained product was separated into chiral enantiomers in the presence of N-Boc. Finally, after providing a free amine chloride in HCl, it was concentrated with an intermediate containing carboxylic acid.^134^

##### Zavegepant

**Figure 15:**
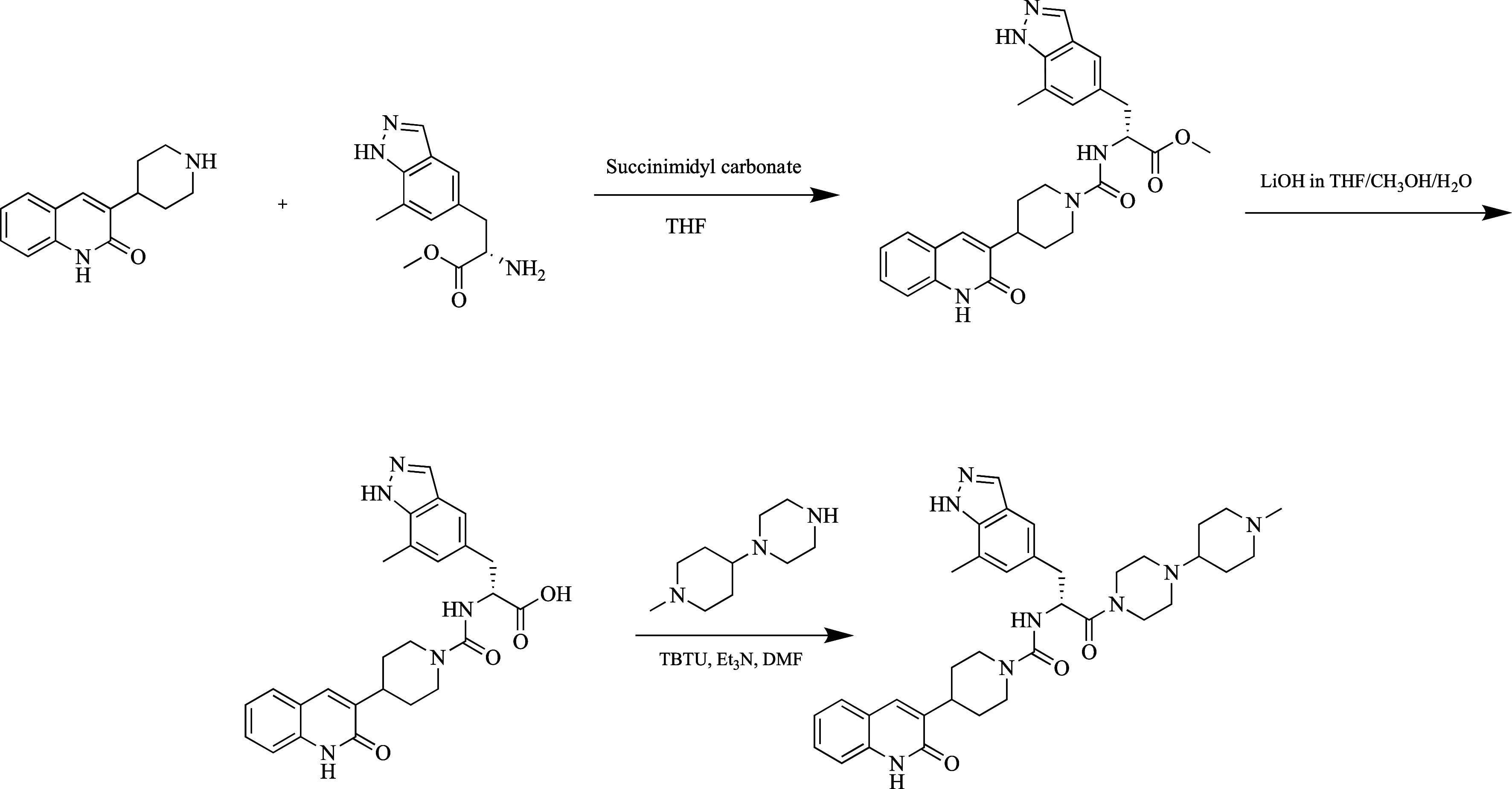
Synthesis of zavegepant.

Zavegepant was the first intranasal spray approved by the FDA for the treatment of acute and chronic migraine. Its superiority over oral gepants was advantageous for those with severe nausea. It started to be prescribed in the U.S. in July 2023. It relieves pain in 15 min and shows its effectiveness even at 10 mg. It is a potent and selective drug with improved oxidative stability.^131^ Phase IIb/III studies have been completed, and no evidence of hepatotoxicity has been observed.^45,137^ Due to its high water solubility, poor permeability, and low oral bioavailability, the intranasal formulation was developed.^138^ Its synthesis is shown in Figure 15.

The starting compounds were bridged with *N,N*′-disuccinimidyl carbonate to form the urea chain. The carboxylic acid-containing intermediate formed after hydrolysis of the methyl group at the ester group was combined with 1-(1-methylpiperidin-4-yl)piperazine.^139^

### 6.4 Summary

The FDA-approved drugs for acute and preventative treatments of migraine discussed above are summarized in Table 2.

**Table 2:** FDA-Approved Drugs for Acute and Preventive Treatments of Migraine

Drug	Mechanism	Treatment	Dosage	ADR	FDA Approval Date
Amytriptilin	suppress cortical spreading depression	preventive	10–75 mg daily, max 25 weeks	hepatotoxicity	1961
Ergotamine	selective agonist of 5-HT1D receptors	acute	max dose 6 mg/day	vomiting and nausea	1976
Propanolol	β-blocker	preventive	40–240 mg daily	dizziness, tiredness	1987
Flunarizine	increases the threshold for cortical spreading depression	preventive	5–10 mg	weight gain, fatigue and drowsiness	1980
Topiramate	GABAergic inhibition, blocking excitatory ion channels	preventive	50–200 mg once or twice	paresthesia, dysgeusia	1996
Valproate	GABAergic inhibition, blocking excitatory ion channels	preventive	500–2000 mg	teratogenic	1996
Dihydroergotamine	selective agonist of 5-HT1D receptors	acute	0.3–1 mg/10 mg metoclopramide every 8 hours for 2–3 days (IV)	nausea	1997

Analgesics					
Acetaminophen/aspirin/caffein	multimechanism	acute	250/250/65 mg	stomach, abdominal pain	1998
Ibuprofen	non-selective COX inhibition	acute	200–800 mg	dizziness	2006
Naproxen/sumatriptan	non-selective COX inhibition	acute	500/85 mg naproxen/sumatriptan	irregular heartbeat	2008
Diclofenac	non-selective COX inhibition	acute	50–100 mg	nausea	2009

Triptans					
Sumatriptan	non-selective agonist of 5-HT1B/1D receptors	acute	max 300 mg tablets, nasal spray two, two injections dose oral and nasal	cardiovascular symptoms	1992
Zolmitriptan	non-selective agonist of 5-HT1B/1D receptors	acute	2.5–5 mg (oral and nasal)	cardiovascular symptoms	1997
Naratriptan	non-selective agonist of 5-HT1B/1D receptors	acute	10 mg oral	cardiovascular symptoms	1998
Rizatriptan	non-selective agonist of 5-HT1B/1D receptors	acute	5–10 mg oral	cardiovascular symptoms	1998
Almotriptan	non-selective agonist of 5-HT1B/1D receptors	acute	12.5 mg	drowsiness, dizziness	2001
Frovatriptan	non-selective agonist of 5-HT1B/1D receptors	acute	2.5 mg	cardiovascular symptoms	2001
Eletriptan	non-selective agonist of 5-HT1B/1D/1F receptors	acute	40 mg	coronary arterial disease	2002

Monoclonal Antibodies					
Erenumab	CGRP receptor blocker	preventive	70–140 mg	nausea, fatigue	2018
Galcanezumab	CGRP receptor blocker	preventive	120 mg/month dose after 240 mg loading dose	constipation	2018
Fremanezumab	CGRP receptor blocker	preventive	225 mg subcutaneously every month or 675 mg every 3 months	swelling at the injection site	2018
Eptinezumab	CGRP receptor blocker	preventive	100 mg IV every 3 months	upper respiratory tract infection	2020

Gepants					
Ubrogepant	CGRP receptor antagonist	acute	max 200 mg per 24 h	nausea	2019
Rimegepant	CGRP receptor antagonist	acute, preventive	75 mg	none	2020
Atogepant	CGRP receptor antagonist	preventive	10–120 mg, 12 weeks	none	2021
Zavegepant	CGRP receptor antagonist	acute	10 mg (nasal spray)	none	2023

Ditans					
Lasmiditan	selective 5-HT1F receptor agonist	acute	50–100 mg	dizziness	2019

## 7 Conclusion

Since many people in the world suffer from migraine disease, the need to understand the mechanism and treatment of migraine is increasing day by day. For this reason, many new drugs such as triptans, gepants, and ditans have been discovered in recent years in addition to the known analgesics for the relief of symptoms such as headache that occur during a migraine attack. Each year the aim is to reduce the side effects caused by the previous generation of drugs, especially vasoconstriction and liver toxicity. Although it is known that migraine pain has trigeminovascular origin, the exact mechanism of migraine is still unknown. The main pathway for discovering new drugs is based on understanding the pathophysiology of migraine. Once the precise mechanism of migraine is discovered, it is thought that significant success in targeted therapies can be achieved in the future.
